# Editorial: Metabolic reprogramming in cancer

**DOI:** 10.3389/fphar.2025.1583986

**Published:** 2025-03-11

**Authors:** Valentina Audrito, Elisa Giovannetti

**Affiliations:** ^1^ Department of Science and Technological Innovation, University of Eastern Piedmont, Alessandria, Italy; ^2^ Department of Medical Oncology, Lab of Medical Oncology, Cancer Center Amsterdam, Imaging and Biomarkers, Amsterdam UMC, Vrije Universiteit, Amsterdam, Netherlands; ^3^ Cancer Pharmacology Lab, Fondazione Pisana Per La Scienza, Pisa, Italy

**Keywords:** metabolic reprogramming, cancer, tumor microenvironment, metabolic pathways, metabolites, tumorigenesis, metabolic plasticity

Technological advancements over the past few decades have unraveled the diversity and adaptability of tumors, shedding light on key genetic aberrations and metabolic pathways that support tumor growth. Specifically, cancer cells alter their metabolic pathways to fulfill the augmented energy and building block requirements while managing oxidative stress crucial for their proliferation and survival (Nong et al., 2023). The flux through these metabolic pathways, underlying of cancer metabolic plasticity, is controlled by cancer driver mutations and environmental nutrient availability.

The tumor microenvironment (TME), often deficient in specific nutrients, compels cancer cells to adapt by inducing mechanisms to scavenge nutrients and sustain their proliferation. Moreover, it is increasingly recognized that the metabolism of non-cancerous cell types within the TME, such as endothelial cells, fibroblasts, and immune cells, can influence tumor progression (Xia et al., 2021). Specifically, metabolic reprogramming is also essential for maintaining self and body homeostasis of various types of immune cells. Recent studies have highlighted that immune cells undergo metabolic reprogramming during proliferation, differentiation, and execution of effector functions, which are crucial for regulating the antitumor immune response (Hu et al., 2024). This impact is achieved by the release of metabolites and its effects on the expression of immune molecules. Considering that metastases are a significant cause of cancer-related deaths, ongoing efforts focus on comprehending how metabolism is employed by metastatic cells, especially in aggressive tumor types such as lung and pancreatic cancers (Comandatore et al., 2022). Furthermore, there is a newfound interest in utilizing cancer genetic analysis to stratify patients and design dietary interventions along with metabolism-targeting therapies.

This Research Topic included 12 original and review papers addressing different features of metabolic reprogramming in tumors, offering novel knowledge on this topic, also at a translational point of view.

In their review article Chen et al. summarized the main features of metabolic reprogramming in tumors, addressing different aspects including increased glycolytic metabolism, lipid synthesis, alteration in amino acids production, and the relationship between altered metabolism and immune response. Then, they focused the paper on the roles played by metabolic adaptation mechanisms in the prognosis and progression of kidney cancer, discussing recent advancements in the diagnosis and treatment of renal cancer targeting metabolic vulnerabilities. The role of metabolic reprogramming was also emphasized in the systematic review by Li et al. analyzing hepatocellular carcinoma (HCC), a cancer with high morbidity and mortality. The authors selected from 2011 to 2023 a total of 575 publications on this field to identify the hotspots and frontiers of metabolic reprogramming research in HCC and to provide future directions for novel scientific research and decision-making in HCC therapeutic strategies.

In the context of metabolic rewiring serine hydroxymethyltransferases (SHMTs) and methylenetetrahydrofolate dehydrogenases (MTHFDs) are recognized as important one-carbon metabolic enzymes for regulating tumor initiation and development, representing potential therapeutic targets for anti-tumor strategies, as illustrated in Zhang et al. MTHFD1/2 have been identified as oncogenic enzymes upregulated in various tumors, involved in metastasis formation and chemoresistance. Cytoplasmic SHMT1 and mitochondrial SHMT2 provide one-carbon units for nucleotide biosynthesis, regulating DNA methylation and NADPH generation, altered during cancer development. Wang et al. discuss how esophageal squamous cell carcinoma (ESCC) cells adapt to a hypoxic, nutrient-deprived microenvironment by rewiring glucose, lipid, and amino acid metabolism. This metabolic shift ensures survival and proliferation despite adverse conditions, highlighting new avenues for therapeutic intervention. The study identifies metabolic vulnerabilities in ESCC, suggesting that disrupting these adaptive pathways could improve treatment efficacy.

Xie et al. further explore how hypoxia-related genes influence prognosis and immunotherapeutic outcomes in ESCC. They establish an HPRscore based on hypoxia phenotype-related genes, demonstrating its predictive power for patient survival and response to treatment. Notably, the study identifies PKP1 as a potential therapeutic target, showing that its knockdown reduces tumor proliferation and migration. These findings provide valuable insight into how hypoxia-driven metabolic changes affect tumor behavior and immune evasion. Peppicelli et al. focused their research on melanoma cells resistant to *anoikis*, to investigate the metabolic reprogramming within circulating tumor cells (CTCs), with the aim of identifying new metabolic targets of CTCs. They discovered that *anoikis*-resistant melanoma cells in suspension show a metabolic rewiring from a characteristic glycolytic pathway toward a more oxidative metabolism based on the use of glutamine and fatty acids, while re-adhesion of CTCs on the dishes reversed the metabolism to glycolysis. The inhibition of the metabolic switch of CTCs led to a reduction of cell viability and colony formation ability of cells capable of surviving in suspension, offering novel and future strategies of treatment of CTCs and melanoma metastases. Similar metabolic adaptations are evident in colorectal cancer (CRC), where immune evasion is closely tied to metabolic shifts in the tumor microenvironment. Nicolini et al. examine how CRC cells undergo metabolic reprogramming—from enhanced glycolysis to increased lipid synthesis—to create an immunosuppressive microenvironment. They discuss how lactate acidification, driven by the Warburg effect, impairs anti-tumor immune cells and promotes tumor-associated macrophages (TAMs) and regulatory T cells (Tregs). The study also explores the role of genetic mutations (e.g., RAS, EGFR) and microbiota in shaping CRC metabolism, emphasizing the potential for metabolic-targeted therapies in combination with immune checkpoint inhibitors (ICIs). Gao et al. provide a broader perspective on metabolic reprogramming as a key tool for predictive and precision medicine. They highlight how different cancer types rely on distinct metabolic adaptations, necessitating tailored therapeutic approaches. The study underscores the growing importance of metabolic profiling in developing personalized treatments, optimizing immune responses, and overcoming drug resistance.

Ferroptosis, a non-apoptotic form of cell death driven by iron-dependent lipid peroxidation, plays a paradoxical role in cancer. Complex metabolic changes within tumor cells and in the tumor microenvironment further influence the response of tumor cells to ferroptosis. As explored by Zhao et al. ferroptosis can both suppress and promote tumor growth depending on cellular context and regulatory mechanisms. The study highlights potential therapeutic strategies for enhancing ferroptosis sensitivity in cancer cells while also addressing resistance mechanisms. Understanding ferroptosis is critical for optimizing cancer treatments, particularly in combination with immunotherapies and metabolic interventions. Of note, one driver of ferroptosis is lipid metabolism, which also plays a vital role in cancer stem cell (CSC) maintenance. Du et al. examine how CSCs manipulate lipid metabolism to sustain their stemness, resist therapy, and adapt to environmental stress. They describe how CSCs increase fatty acid content for energy, engage in β-oxidation to optimize utilization, and enhance cholesterol synthesis through the mevalonate pathway. Additionally, lipid droplets serve as alternative energy reservoirs, protecting CSCs from oxidative stress. This study underscores the need to target lipid metabolism to weaken CSC resilience and improve treatment outcomes. Lastly, Ping et al. in their original article using a retrospective analysis described the predictive value of altered plasma omega-3 polyunsaturated fatty acids (omega-3 PUFAs) levels for early treatment response, progression free survival, and overall survival in patients with cervical squamous cell carcinoma (CSCC) who underwent concurrent chemoradiotherapy (CCRT). Interestingly, the authors demonstrated that pretreatment plasma omega-3 PUFAs level, may be a promising biomarker for predicting recent response in CSCC, increasing the prognostic significance of serum squamous cell carcinoma antigen (SCC)-Ag level alone, opening to create new prognostic tools for clinicians in CSCC.

Together, these studies reveal how metabolic reprogramming drives tumor growth and progression, contributes to metastasis formation and chemoresistance, and is interconnected with ferroptosis and lipid metabolism. Targeting these metabolic vulnerabilities holds great promise for improving cancer treatment, particularly in combination with immunotherapy and precision medicine strategies.
